# Supplementary material for: Characterization of the Brassica napus Flavonol Synthase Gene Family Reveals Bifunctional Flavonol Synthases
Source: Front Plant Sci. 2021 Oct 13;12:733762. doi: 10.3389/fpls.2021.733762 (PMC8548573; doi:10.3389/fpls.2021.733762)
Supplement: Supplementary file 6 [file Data_Sheet_1.PDF]

Supplementary Material

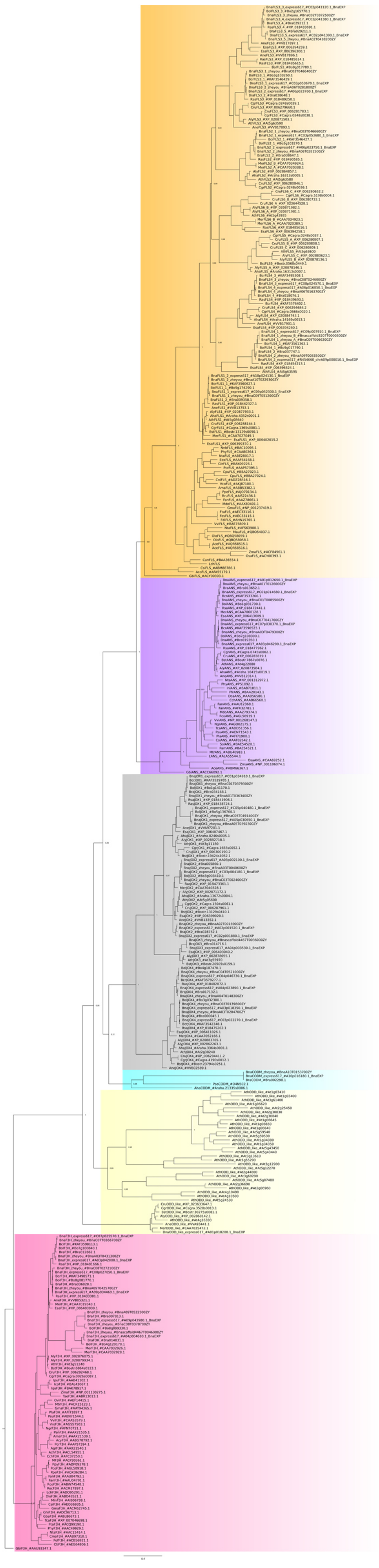

Supplementary Figure S1: Phylogeny of BnaFLS candidates and other plant 2-ODDs. Relative bootstrap-values are shown next to relevant nodes. The phylogenetic tree is based on amino acid sequences of the 2-ODD members FLS, ANS, F3H, and 2-ODD-like sequences derived from Kawai *et al.* 2014.

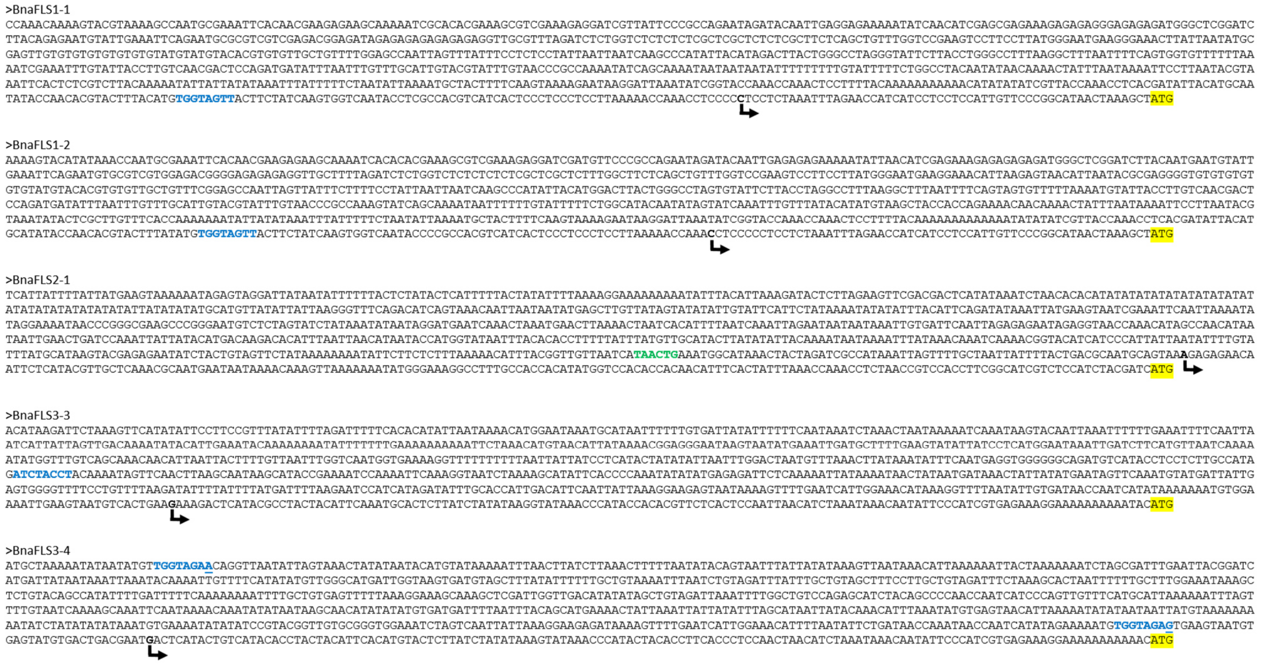

Supplementary Figure S2: MYB recognition elements of *BnaFLSs*.
MYB recognition elements upstream of the transcriptional start site (black arrow) annotated based on newly generated RNA-Seq data of *BnaFLS* genes are shown. The SG7 consensus MRE (5'-AcCTACCa-3'/5'-tGGTAGgT-3') is marked in blue, while the MRE of MYB24 (5'-CNGTTR-3'/5'-RAACNG-3') is shown in green. Bases differentiating from the consensus motif are underlined. The start codon is highlighted in yellow.

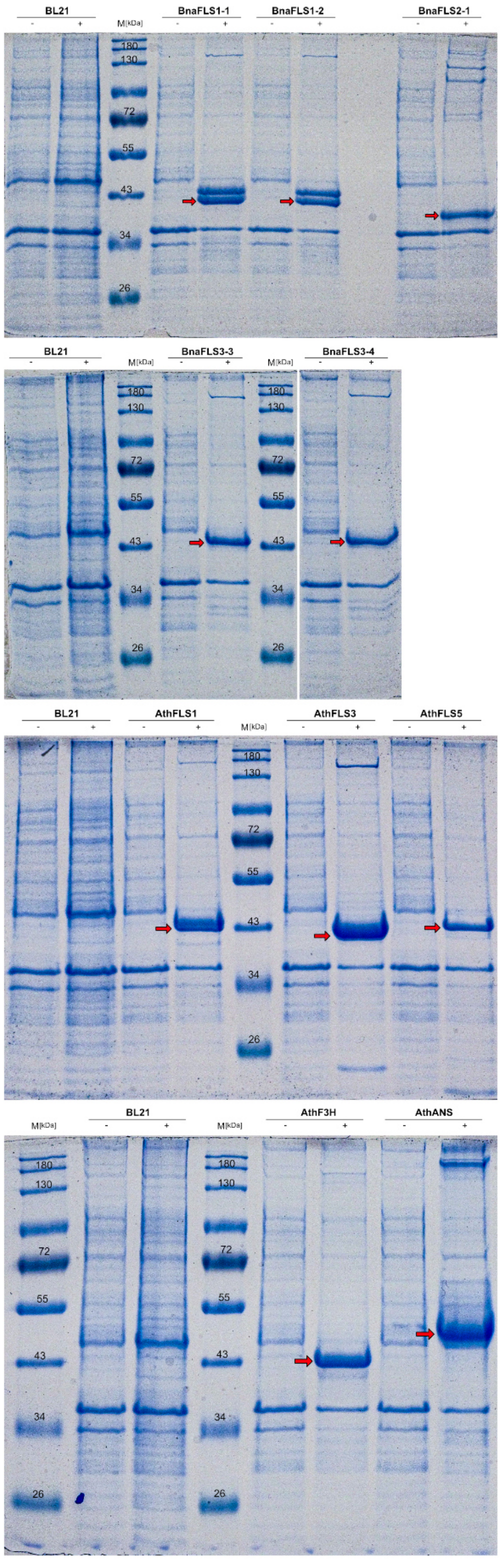

Supplementary Figure S3: SDS-PAGE of recombinant proteins analyzed in this work. Recombinant proteins are marked with a red arrow. The *E. coli* strain BL21 was used as control. uninduced (-), induced (+).

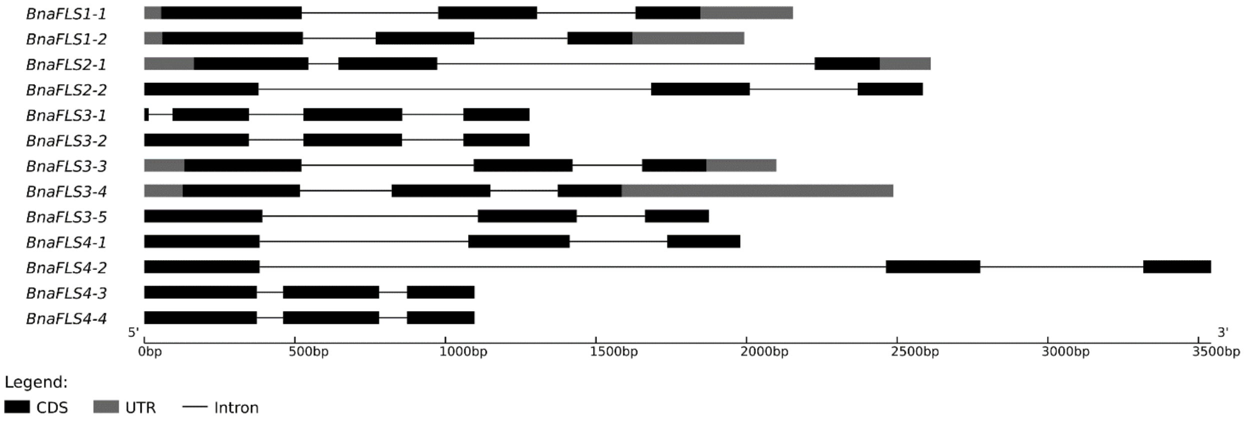

Supplementary Figure S4: Genomic structure of *BnaFLSs*.
The exon-intron structure of *BnaFLSs* is shown. The exons are split into coding sequences (CDS, black) and untranslated regions (UTR, gray) and are displayed by rectangles, introns are displayed as black connecting lines.

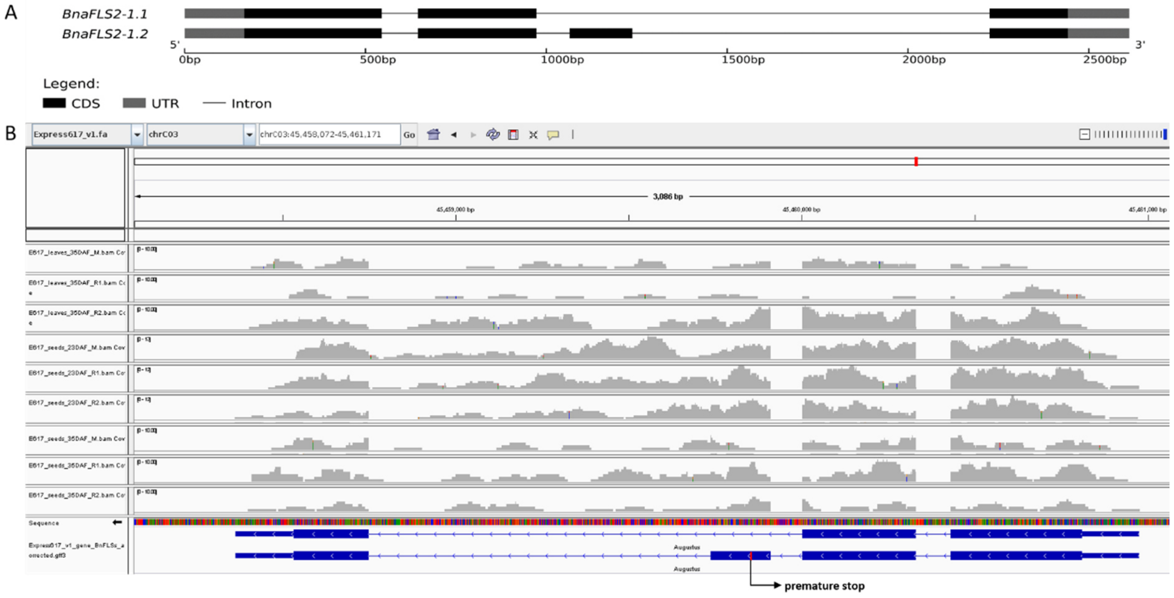

Supplementary Figure S5: Genomic structure of the alternative transcript of *BnaFLS2-1.2*. (A) The exon-intron structure of *BnaFLS2-1.1 and BnaFLS2-1.2* is shown. The coding sequences (CDS, black), untranslated regions (UTR, gray), and introns are displayed by black and gray rectangles, as well as black connecting lines, respectively. The alternative transcript *BnaFLS2-1.2* contains an additional third exon of 173 bp rendering the encoded 253 amino acid protein most likely non-functional. (B) A frameshift causes a nonsense mutation in the additional third exon. This transcript was observed in seed samples (23 DAF and 35 DAF).

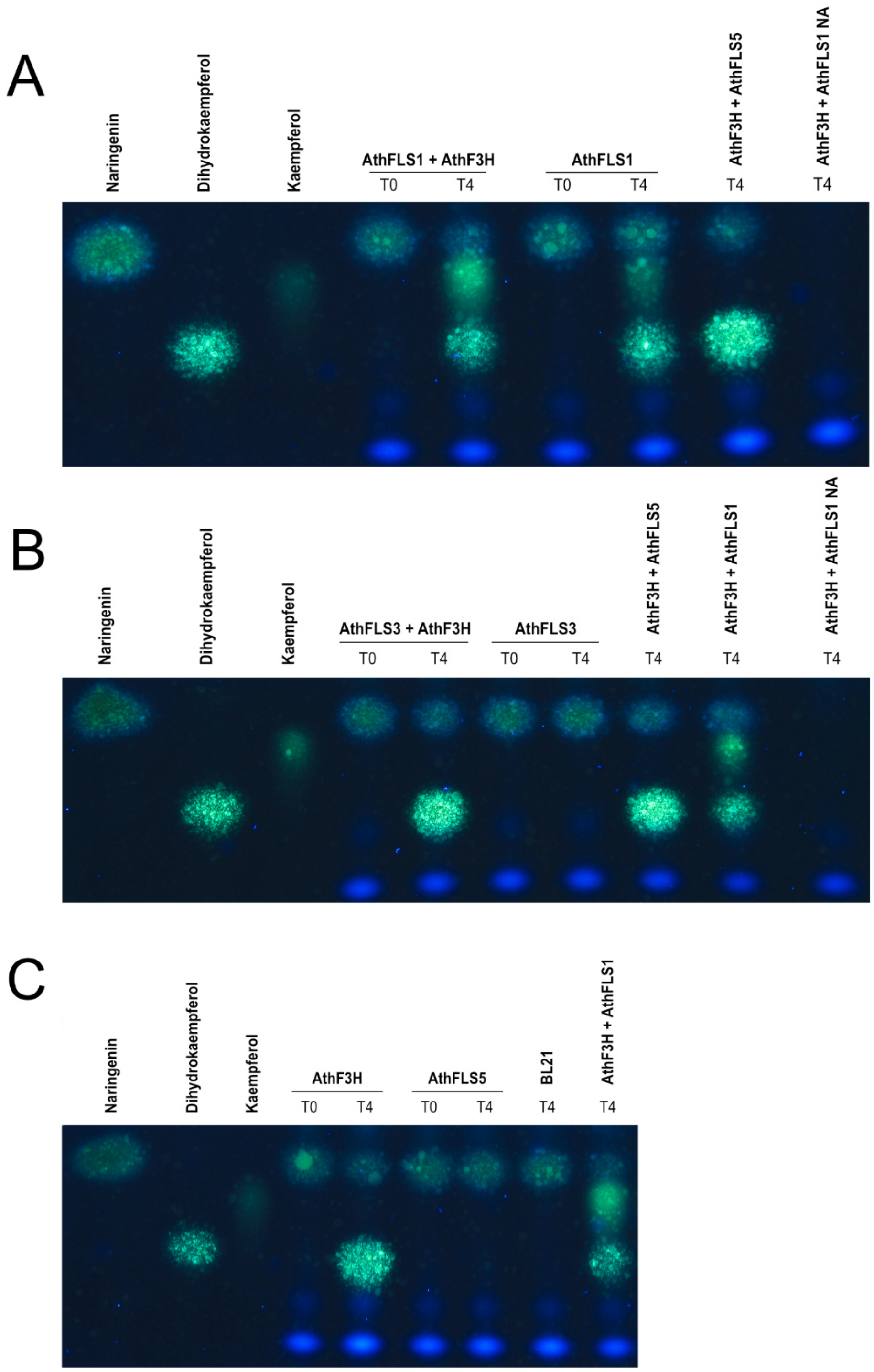

Supplementary Figure S6: Bioconversion assays of *A. thaliana* 2-ODD members.
(A) and (B) Bioconversion assay results based on a HPTLC using extracts from *E. coli* expressing recombinant AthFLS1 or AthFLS3, respectively. The substrate of F3H naringenin, as well as the FLS substrate dihydrokaempferol and the product kaempferol were used as standards. AthFLS1 served as positive control and AthFLS5 as negative control. In the last sample no Nargingenin (NA) was supplemented. (C) Bioconversion assay results of AthF3H and AthFLS5. The *E. coli* strain BL21 was used as control.

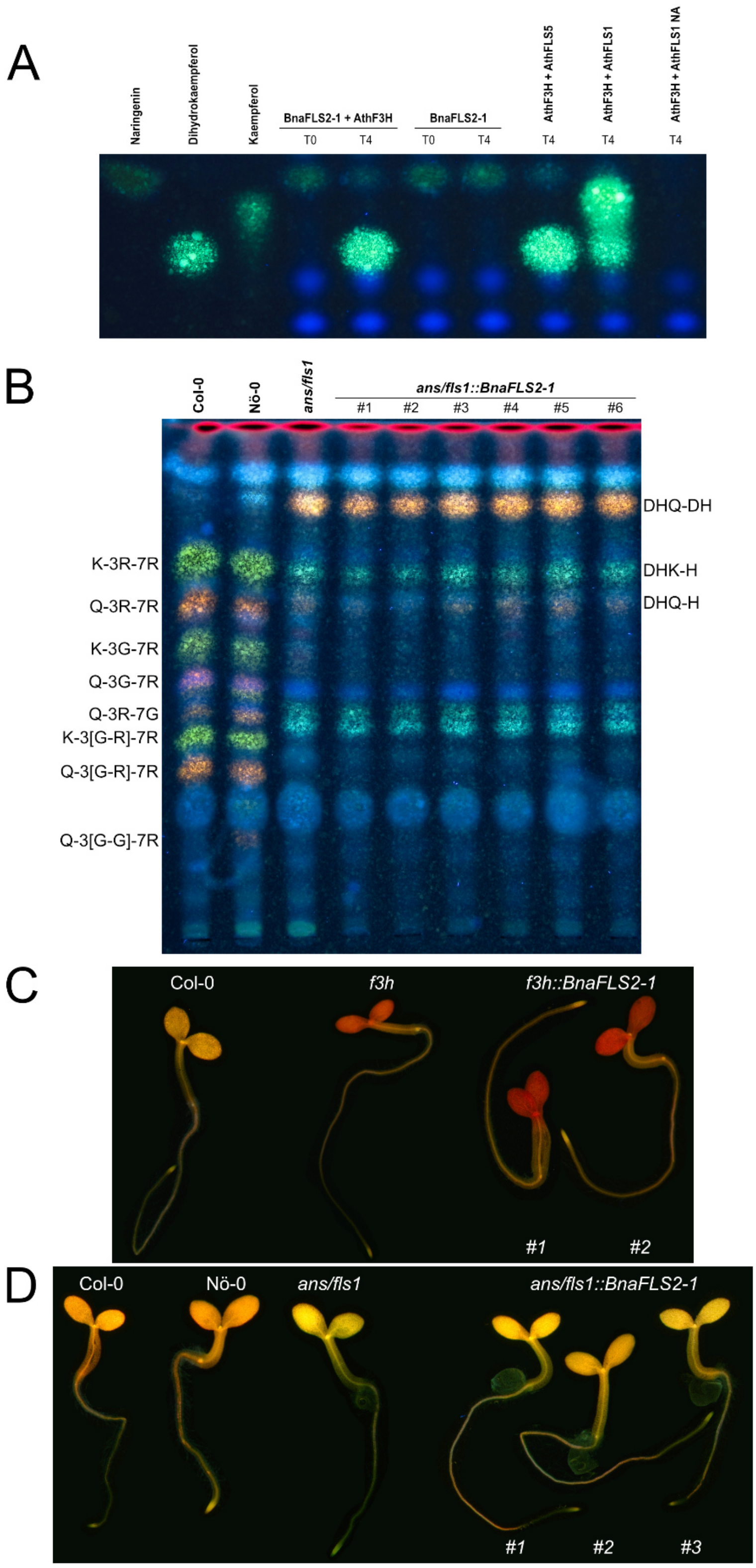
A
Naringenin
Dihydrokaempferol
Kaempferol
BnaFLS2-1 + AthF3H
BnaFLS2-1
AthF3H + AthFLS5
AthF3H + AthFLS1
AthF3H + AthFLS1 NA
T0
T4
B
Col-0
Nö-0
ans/fls1
ans/fls1::BnaFLS2-1
#1
#2
#3
#4
#5
#6
K-3R-7R
Q-3R-7R
K-3G-7R
Q-3G-7R
Q-3R-7G
K-3[G-R]-7R
Q-3[G-R]-7R
Q-3[G-G]-7R
DHQ-DH
DHK-H
DHQ-H
C
Col-0
f3h
f3h::BnaFLS2-1
#1
#2
D
Col-0
Nö-0
ans/fls1
ans/fls1::BnaFLS2-1
#1
#2
#3

Supplementary Figure S7: Functional characterization of BnaFLS2-1.
See Figure 5 for detailed figure description. (A) Bioconversion assay results of BnaFLS2-1. (B) The following flavonoid derivates were additionally labeled: dihydroquercetin-deoxyhexoside (DHQ-DH), dihydrokaempferol-hexoside (DHK-H), dihydroquercetin-hexoside (DHQ-H), quercetin-3-O-rhamnoside-7-O-glucoside (Q-3R-7G). (C) and (D) Flavonol staining in young seedlings.
