# Supplementary material for: Characterization of the Brassica napus Flavonol Synthase Gene Family Reveals Bifunctional Flavonol Synthases
Source: Front Plant Sci. 2021 Oct 13;12:733762. doi: 10.3389/fpls.2021.733762 (PMC8548573; doi:10.3389/fpls.2021.733762)
Supplement: Supplementary file 7 [file Data_Sheet_6.PDF]

UNIVERSITÄT BIELEFELD

CeBiTec
Center for Biotechnology

Dr. Daniela Holtgräwe
Genetics and Genomics of Plants
CeBiTec (Center for Biotechnology)
Bielefeld University
Sequenz 1
33615 Bielefeld

Dear Editor,
dear editorial team of *Frontiers in Plant Science*,

Please find enclosed our manuscript entitled "**Characterization of the *Brassica napus* flavonol synthase gene family reveals bifunctional flavonol synthases**" by Hanna Marie Schilbert *et al.*, which we are submitting as an original article to *Frontiers in Plant Science*.

We characterized the *Brassica napus* flavonol synthase (FLS) gene family. Starting with a phylogenetic analysis the evolutionary events shaping the *FLS* gene family consisting of 13 members are discussed. Spatio-temporal transcription patterns across various organs revealed the major *FLS* genes expressed in seeds, the main organ of agronomical importance. Of these *FLS* genes, two *BnaFLS1* homoelogs were identified to encode bifunctional enzymes, exhibiting FLS and F3H activity. Both *BnaFLS1* homoelogs were found to be co-expressed with major players of the flavonoid biosynthesis. Bifunctionality of FLS has not yet been reported for *B. napus*. Finally, potential mechanisms underlying bifunctionality are discussed.

This manuscript has not been submitted to any journal previously and is not under consideration for publication at any other place. Since *Frontiers in Plant Science* frequently publishes papers touching on the flavonoid biosynthesis, we consider it an excellent fit for our manuscript.

Yours sincerely,

Daniela Holtgräwe (as corresponding author for all authors)
